# Microvascular ischemia in hypertrophic cardiomyopathy: new insights from high-resolution combined quantification of perfusion and late gadolinium enhancement

**DOI:** 10.1186/s12968-016-0223-8

**Published:** 2016-01-14

**Authors:** Adriana D. M. Villa, Eva Sammut, Niloufar Zarinabad, Gerald Carr-White, Jack Lee, Nuno Bettencourt, Reza Razavi, Eike Nagel, Amedeo Chiribiri

**Affiliations:** 1 Division of Imaging Sciences, King’s College London, Wellcome Trust/EPSRC Medical Engineering Centre, St Thomas’ Hospital, London, UK; 2 Cardiology Department, St Thomas’ Hospital, London, UK; 3 Hospital Center Vila Nova Gaia, Porto, Portugal; 4 DZHK Centre for Cardiovascular Imaging, University Hospital Frankfurt / Main, Frankfurt am Main, Germany; 5 Division of Imaging Sciences and Biomedical Engineering, King’s College London, The Rayne Institute – 4th Floor Lambeth Wing, St Thomas’ Hospital, SE1 7EH London, UK

**Keywords:** Hypertrophic cardiomyopathy, Stress test, Perfusion imaging, Adenosine, Late gadolinium enhancement, Cardiovascular magnetic resonance

## Abstract

**Background:**

Microvascular ischemia is one of the hallmarks of hypertrophic cardiomyopathy (HCM) and has been associated with poor outcome. However, myocardial fibrosis, seen on cardiovascular magnetic resonance (CMR) as late gadolinium enhancement (LGE), can be responsible for rest perfusion defects in up to 30 % of patients with HCM, potentially leading to an overestimation of the ischemic burden. We investigated the effect of left ventricle (LV) scar on the total LV ischemic burden using novel high-resolution perfusion analysis techniques in conjunction with LGE quantification.

**Methods:**

30 patients with HCM and unobstructed epicardial coronary arteries underwent CMR with Fermi constrained quantitative perfusion analysis on segmental and high-resolution data. The latter were corrected for the presence of fibrosis on a pixel-by-pixel basis.

**Results:**

High-resolution quantification proved more sensitive for the detection of microvascular ischemia in comparison to segmental analysis. Areas of LGE were associated with significant reduction of myocardial perfusion reserve (MPR) leading to an overestimation of the total ischemic burden on non-corrected perfusion maps. Using a threshold MPR of 1.5, the presence of LGE caused an overestimation of the ischemic burden of 28 %. The ischemic burden was more severe in patients with fibrosis, also after correction of the perfusion maps, in keeping with more severe disease in this subgroup.

**Conclusions:**

LGE is an important confounder in the assessment of the ischemic burden in patients with HCM. High-resolution quantitative analysis with LGE correction enables the independent evaluation of microvascular ischemia and fibrosis and should be used when evaluating patients with HCM.

## Background

Hypertrophic cardiomyopathy (HCM) is the most common genetic cardiomyopathy [1]. While the majority of patients remain asymptomatic, in a subset of patients the prognosis is poor with progression to heart failure or presentation with sudden cardiac death (SCD) [2–4]. HCM is considered the most common cause of SCD in young competitive athletes [5, 6].

The hallmarks of the disease include left ventricle (LV) hypertrophy, fibrosis and microvascular ischemia [7] and it remains debated as to whether there is a causative link between these features [8]. Microvascular ischemia can be diagnosed using non-invasive imaging modalities, including positron emission tomography (PET) [7], single photon emission computed tomography (SPECT) [9] and first-pass perfusion cardiovascular magnetic resonance (CMR) [10, 11]. CMR is emerging as the imaging modality of choice for HCM due to its unrivalled capability to assess LV hypertrophy and LV fibrosis. Alongside structural changes, CMR can also assess the presence of ischemia within a single examination. To date however, only two studies have used CMR to evaluate the relationship between LV hypertrophy, fibrosis and perfusion [10, 12]. The study from Petersen et al. [10] used segmental quantitative perfusion analysis, while Ismail et al. [12] demonstrated the feasibility of high-resolution perfusion quantification in patients with HCM. Both studies proved a close relationship between the severity of microvascular ischemia, the degree of hypertrophy and the severity of LV fibrosis in keeping with previous studies that used PET [7, 13, 14], or a combination of PET and CMR [15]. However, no studies have specifically explored the impact of late gadolinium enhancement (LGE) on perfusion analysis in HCM. This is of particular relevance since LGE is observed in 60-80 % of patients and is frequently associated with rest perfusion abnormalities [16–19].

High-resolution quantification of perfusion CMR has been shown to provide accurate perfusion estimates [20, 21] and, in contrast to segmental quantification, preserves the spatial resolution of the original imaging data, potentially resulting in a more sensitive detection of microvascular ischemia [12]. Another possible and yet unexplored advantage of high-resolution perfusion quantification is the possibility to combine the interpretation of quantitative perfusion results in conjunction with the results of LV scar analysis on a high-resolution level.

The aim of this study is to demonstrate the feasibility of combined high-resolution quantitative perfusion and LGE analysis. Specifically, we aimed to determine the effect of overt LV scar on the total LV ischemic burden.

## Methods

Consecutive patients with a clinical diagnosis of HCM and visually unobstructed or with minor non-obstructive atheroma (30 % visual stenosis or less) on invasive coronary angiography referred for perfusion CMR were retrospectively identified. The diagnosis of HCM was based on confirmation from genetic testing or on conventional criteria for HCM diagnosis (presence of a wall thickness ≥ 15 mm, or 13 mm in patients with family history of HCM, without chamber dilation and in the absence of any other systemic or cardiac disease sufficient to justify the hypertrophy). Patients had been scanned on clinical grounds at St. Thomas’ Hospital, London. All patients gave written and informed consent at the time of the scan (ethics committee approval 15/NS/0030). This study was performed in accordance with the principles of the Declaration of Helsinki.

Scans were performed at 3.0 T (Philips Achieva-TX, Philips Medical Systems) and included myocardial function, stress and rest perfusion and late gadolinium enhancement imaging using standard acquisition protocols [22]. A 32-channel cardiac phased array receiver coil was used for all studies.

The perfusion sequences were performed during adenosine-induced hyperemia over 3 min (140 μg/kg/min) and repeated 15 min later at rest, both times using 0.075 mmol/kg gadobutrol (Gadovist, Bayer, Berlin, Germany) at 4 ml/s followed by a 20 ml saline flush. A dual bolus protocol for contrast agent injection was used as previously described [23]. All subjects were asked to abstain from caffeine and caffeine-containing food and drinks for at least 24 hours before the scan, according to institutional practice.

First-pass perfusion imaging consisted of a high-resolution *kt* turbo-gradient echo sequence [typical imaging parameters: shortest echo time (range 1.35–1.54 ms), shortest repetition time (range 2.64–3.12 ms), 18° flip angle, 90° saturation prepulse, 120 ms prepulse delay, typical TR 2.6 ms, typical TE 0.9 ms, typical spatial resolution 1.2x1.2x10mm)]. Three short-axis slices (basal, mid and apical) were acquired over every heartbeat covering 16 of the standard myocardial segments (segment 17 was excluded). A correction map was created from a proton density-based image based on the same projections as the perfusion scans for correction of spatial inhomogeneities due to surface coils [24]. LGE imaging was acquired after a top up dose of contrast agent to a total dose of 0.2 mmol of gadolinium/kg of body weight, according to standard acquisition methods [22].

### Image analysis

Image analysis was performed according to standard practice [25]. Perfusion series were evaluated visually for the presence or absence of perfusion abnormalities. Quantitative perfusion analysis was performed both on a segmental and high-resolution basis. High-resolution results were evaluated with and without inclusion of regions with overt LGE, identified using a standard threshold-based approach as detailed below.

#### Standard evaluation of the left ventricle

LV function, global LV mass and maximal segmental LV wall thickness were analyzed using commercially available software (CVI42, version 4.1.8, Circle Cardiovascular Imaging Inc., Calgary, Alberta, Canada) according to standardized methods [25]. All the indices were corrected for body surface area. Hypertrophy was defined by an end-diastolic wall thickness equal or greater than 15 mm [1].

Two independent observers judged the presence of fibrosis visually and recorded LGE in terms of standard LV segments. Using commercially-available software, according to standardized methods, areas of scar were measured [26] (CVI42, version 4.1.8, Circle Cardiovascular Imaging Inc., Calgary, Alberta, Canada). A threshold of six standard deviations above the average signal of a remote and non-enhanced region was used to define overt scar [26].

#### Visual perfusion assessment

Perfusion data were assessed visually by two operators by consensus and abnormalities recorded in terms of standard LV segments. Perfusion abnormalities were defined as a visual reduction in the signal intensity (SI) of an area of myocardium lasting longer than five cardiac cycles and not related to obvious respiratory, motion or dark rim artifact.

Perfusion abnormalities were classified visually in three categories: 1) subendocardial perfusion abnormalities, seen as a gradient of reduced perfusion arising from the subendocardium in absence of LGE in the same segment; 2) LGE-related perfusion abnormalities, seen only in the mid-myocardial layers and exclusively in correspondence with overt, confluent areas of LGE; 3) mixed perfusion abnormalities, in segments where both LGE-related and subendocardial perfusion abnormalities were observed.

#### Quantitative perfusion analysis

Two experienced operators, blinded to results of visual perfusion assessment and other clinical data, performed quantitative analysis using software and methods developed and previously validated by our group against perfusion phantom, PET data and microspheres [21, 27, 28]. Quantitative signal intensity (SI) analysis required accurate respiratory motion correction and myocardial contour delineation. Respiratory motion was corrected using affine image registration by maximization of the joint correlation between consecutive dynamics within an automatically determined region of interest. A temporal maximum intensity projection was calculated to serve as a feature image for an automatic contour delineation method. The operator then manually optimized the automatically generated contours to avoid partial volume effects at the endocardial and epicardial border as previously described [29]. Areas of subendocardial dark-rim artifact occurring at the arrival of the main bolus of contrast agent in the LV were carefully excluded from the segmentation.

Segmental quantitative perfusion analysis was performed using spatially averaged myocardial SI curves according to standard cardiac segmentation [30].

Quantitative perfusion analysis was performed by Fermi deconvolution according to the methods described by Wilke et al. [31] and Jerosch-Herold et al. [32] where time curves for the tissue impulse response function, *h(t)*, were fitted to the Fermi function with the following analytical expression:

$$ h(t)=R\left[\frac{1}{e^{\left(t-{\tau}_0-{\tau}_d\right)k}+1}\right]u\left(t-{\tau}_d\right) $$

using a Marquardt-Levenberg nonlinear least square algorithm by letting *k, R* and *τ*_0_ vary and keeping *τ*_*d*_ fixed. In the above equation, *u*(*t* − *τ*_*d*_) is the unit step function. The *τ*_*d*_ accounts for the delay time between the appearance of the signal in the LV blood pool and myocardial region of interest (ROI) [33] *τ*_0_ characterizes the width of the shoulder of the Fermi function during which little or no contrast agent had left the ROI. *R* is the index of contrast agent influx parameter and *k* represents the decay rate of *h*(*t*) due to contrast agent washout. Using the above equation, myocardial blood flow (MBF) estimates are calculated as *h*(*t*) at *t* = 0 [32].

Myocardial perfusion reserve (MPR) was calculated as the ratio between stress and rest MBF estimates.

#### Combined high-resolution fibrosis and perfusion quantification

Current two-dimensional perfusion techniques involve each slice being acquired in a different phase of the cardiac cycle while LGE is acquired in a pre-specified phase, usually in end-diastole. Therefore, prior to any quantification, the LGE and perfusion images had to be registered. Firstly an LGE slice was selected that matched the corresponding perfusion slice in terms of position within the LV. A pair of endocardial and epicardial LV contours were then drawn on rest and stress perfusion and LGE images and a deformable template segmentation method was applied to the images and optimized using a greedy optimization scheme as previously described [29].

**Fig. 1:**
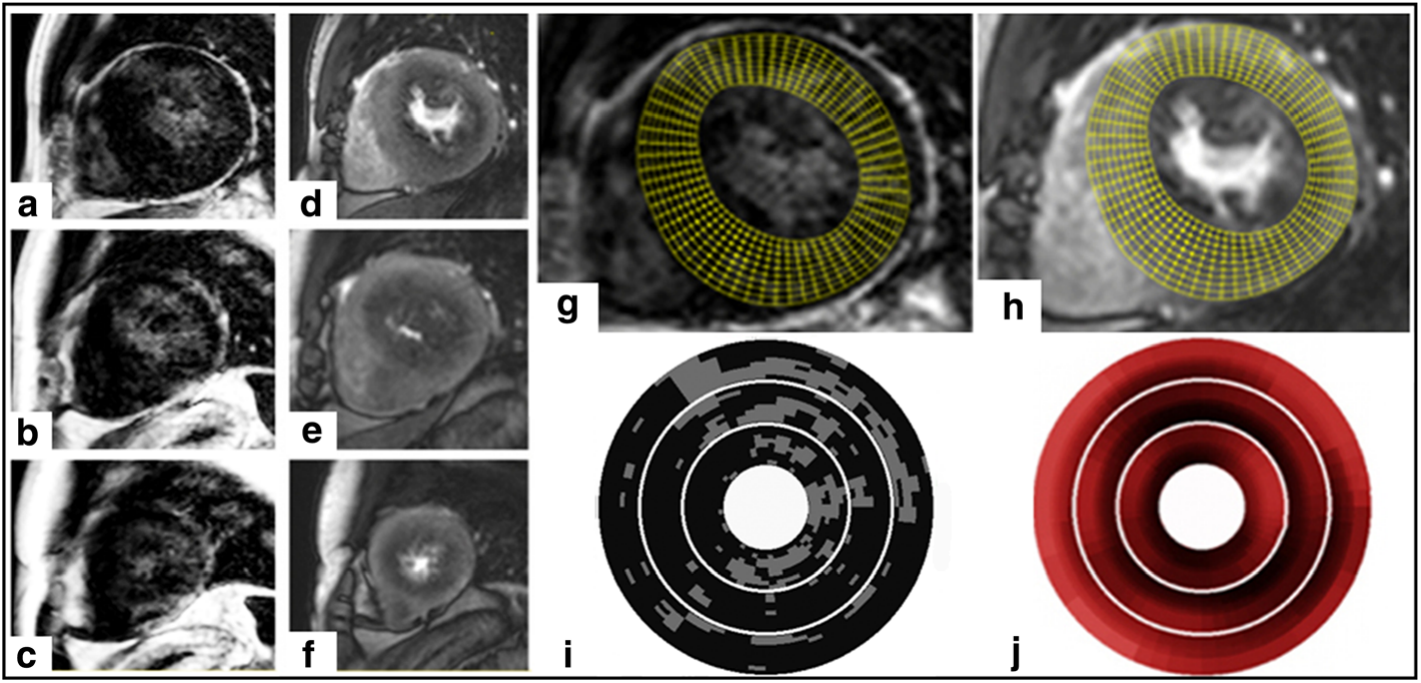
Example of combined high-resolution fibrosis and perfusion mapping. **a**-**c** late gadolinium enhancement (LGE) images. **d**-**f** stress perfusion images. Top, middle and bottom rows correspond with basal, mid and apical slices respectively. Images (**g**) and (**h**) indicate high-resolution maps for LGE and stress perfusion respectively (basal slice only), with the grid used for high-resolution maps of LGE (I) and stress perfusion (J) shown below

High-resolution quantitative MPR and fibrosis maps were generated allowing generation of high-resolution MPR maps and calculation of the ischemic burden with and without inclusion of areas of overt myocardial scar (Fig. 1).

#### Total ischemic burden calculation

In order to represent and compare total ischemic burden measurements obtained using segmental and high-resolution quantitative analysis, we adopted the methods described and recommended by the ISCHEMIA Trial expert panel to compare ischemic burden measurements obtained by different imaging modalities [34]. In brief, the ischemic burden was expressed as percentage of LV myocardium for different MPR values for both segmental and high-resolution results.

#### Statistical analysis

Categorical data are presented as numbers and percentages and continuous data are presented as mean ± standard deviation (SD). The normal distribution of high-resolution MPR estimated was tested with P-P plot and a skewness test. Group means were compared using an unpaired Student *t test* and categorical data were compared between groups using the Fisher exact test and Pearson chi-square test, as appropriate. Values of *p* < 0.05 was considered to be statistically significant. ANOVA was used to determine differences between multiple groups. Bonferroni correction was used to account for multiple testing in both segmental and high-resolution quantitative perfusion analysis. Bland-Altman and Pearson’s analysis were used to assess the interoperator reproducibility of the ischemic burden measurements.

## Results

The study cohort comprised 30 patients (77 % male, age range 61 ± 13 years). Demographics of patients are shown in Table 1.

**Table 1:** Baseline characteristics of the patients and cardiac magnetic resonance (CMR) functional results. Patients are classified according to the results of perfusion (PERF) and late gadolinium enhancement (LGE) visual assessment as four groups: *PERF + LGE+* (perfusion abnormality positive, LGE positive), *PERF + LGE-* (perfusion abnormality positive, LGE negative), *PERF-LGE+* (perfusion abnormality negative, LGE positive) and *PERF-LGE-* (perfusion abnormality negative, LGE negative)

	All (*N* = 30)	PERF + LGE+ (*N* = 12)	PERF + LGE- (*N* = 7)	PERF-LGE+ (*N* = 9)	PERF-LGE- (*N* = 2)
Male gender	23 (77 %)	11 (92 %)	5 (71 %)	6 (67 %)	1 (50 %)
Age (years)	61 ± 13	61 ± 10	52 ± 13	65 ± 13	76 ± 13
LA (cm^2^)	23.1 ± 5.6	22.9 ± 4.8	21.3 ± 2.6	22.4 ± 6.9	33.5 ± 0.7
RA (cm^2^)	20 ± 4.1	20.8 ± 4.4	19.6 ± 2.4	19.3 ± 4.6	20 ± 7.1
LV EF (%)	64.8 ± 10	68.1 ± 7	69.4 ± 5.8	57.4 ± 11.1	62.5 ± 20.5
LV EDV index (ml/m^2^)	71.9 ± 23.2	73.5 ± 20.4	63.3 ± 11.5	71.1 ± 23.9	95.5 ± 63.4
LV ESV index (ml/m^2^)	26.5 ± 15.7	24 ± 9.5	19.5 ± 5.6	31.8 ± 19.1	42.3 ± 42.7
LV mass index (g/m^2^)	82.7 ± 32.8	93.7 ± 38.4	65.6 ± 23	80 ± 27.6	86.4 ± 43.4
RV EF (%)	66.7 ± 8.1	69.8 ± 8.8	64.9 ± 7.4	57.4 ± 11.1	61 ± 4.2
RV EDV index (ml/m^2^)	65.3 ± 17.6	68.2 ± 21.9	63.7 ± 9.8	59.8 ± 9.9	79.2 ± 40
RV ESV index (ml/m^2^)	22.4 ± 8.3	21.5 ± 10.7	22.3 ± 5.4	22.1 ± 5.9	30 ± 12.3
Max LV thickness (mm)	25	25	19	20	21
Average LV thickness (mm)	10.5 ± 4	11.3 ± 4.4	9.4 ± 3.3	10.3 ± 4	10.5 ± 3.3
Hypertrophic segments/patient	3.1 ± 2.6	4.3 ± 3.1	1.4 ± 1.1	3 ± 2.3	1.5 ± 0.7
LGE %	7.3 ± 7.4	11.5 ± 8.4	0.7 ± 0.8	8.2 ± 4.6	0.5 ± 0.7
HR (bpm)					
- Rest	72 ± 12	75 ± 12	76 ± 13	66 ± 11	63 ± 11
- Stress	94 ± 10	96 ± 12	96 ± 6	90 ± 11	87 ± 11
BP (mmHg)					
- Rest	135 ± 14/91 ± 10	140 ± 16/78 ± 13	129 ± 9/80 ± 7	135 ± 14/83 ± 6	130 ± 14/88 ± 9
- Stress	132 ± 20/78 ± 12	137 ± 19/77 ± 12	128 ± 22/80 ± 13	129 ± 20/75 ± 7	132 ± 39/88 ± 22

*PERF* perfusion abnormalities, *LGE* late gadolinium enhancement, *LA* left atrium, *RA* right atrium, *LV* left ventricle, *EF* ejection fraction, *EDV* end-diastolic volume, *ESV* end-systolic volume, *RV* right ventricle, LGE: late gadolinium enhancement *HR* heart rate, *BP* blood pressure

### Standard evaluation of the left ventricle

**Fig. 2:**
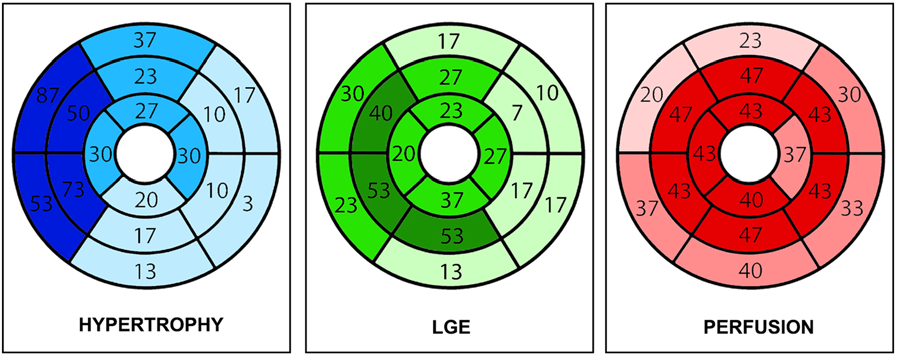
Schematic representation of the distribution of hypertrophic segments, late gadolinium enhancement (LGE) and perfusion abnormalities based on visual assessment, expressed as percentages of the total cohort

Structural and functional CMR findings and hemodynamic parameters are also shown in Table 1. The average number of hypertrophic segments was 3.1 ± 2.6 per patient (range 1–11 segments per patient). Maximum LV wall thickness was >25 mm in 2/30 patients (6 %) and >20 mm in 7/30 patients (23 %). 21/30 patients (70 %) were positive for LV fibrosis, most frequently involving the septal segments. Detailed segmental results of hypertrophy, fibrosis and perfusion are summarized in Fig. 2.

### Visual perfusion assessment

Stress-induced perfusion abnormalities (either subendocardial or mixed) were found in 18/30 (60 %) patients on visual assessment involving 185/304 (61 %) segments amongst positive patients. Subendocardial perfusion abnormalities were seen in 14/30 (47 %) patients and 4/30 (13 %) patients had mixed perfusion abnormalities. 1/30 (3 %) patient had only scar-related perfusion abnormalities. 11/30 (37 %) patients had visually normal perfusion.

No association was observed between average wall thickness of hypertrophic segments and perfusion abnormalities (wall thickness was 16.8 ± 2.1 mm for segments with subendocardial perfusion abnormalities, 15.5 ± 0.7 mm for LGE-related perfusion abnormalities, and 17.6 ± 2.7 mm for mixed perfusion abnormalities; *p* = 0.38).

### Segmental quantitative perfusion

**Fig. 3:**
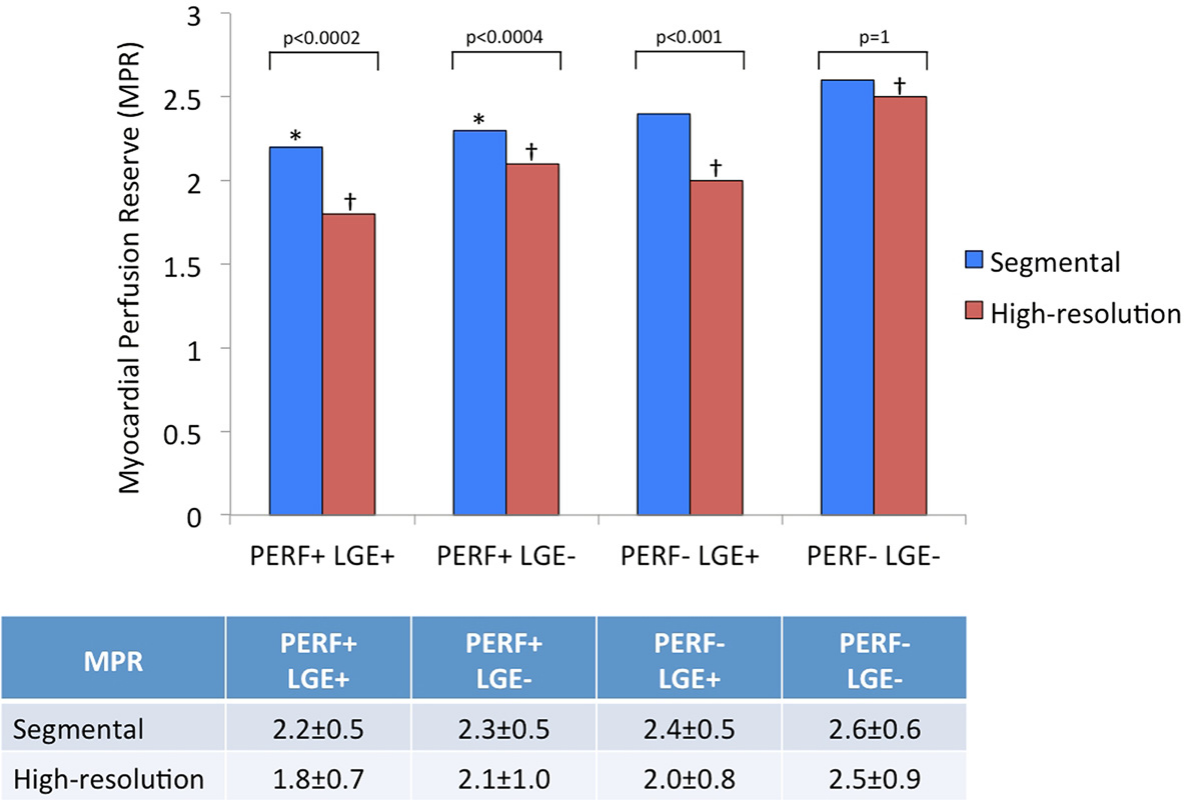
Comparison between myocardial perfusion reserve (MPR) values obtained by using segmental and high-resolution quantification. Regions are classified according to the results of perfusion (PERF) and late gadolinium enhancement (LGE) visual assessment. *p < 0.0001 vs. segmental MPR of PERF-LGE- regions; † *p* < 0.0001 vs. all other groups for high-resolution MPR

Using segmental quantitative analysis, segments with visual perfusion abnormalities and LGE (*PERF + LGE+*) had the lowest MPR values (2.2 ± 0.5). Conversely, segments negative for visual perfusion abnormalities and LGE (*PERF-LGE-*) had the highest MPR values (2.6 ± 0.6). Segments with visual perfusion abnormalities, but no LGE (*PERF + LGE-)* had MPR values of 2.3 ± 0.5. Segments with LGE and visually normal perfusion (*PERF-LGE+*) had MPR values of 2.4 ± 0.5. A significant difference was observed between *PERF + LGE+* versus *PERF-LGE-* (*p* < 0.0001) and *PERF + LGE-* versus *PERF-LGE-* (*p* < 0.0001). Detailed results are presented in Fig. 3.

### High-resolution perfusion quantification

On high-resolution perfusion quantification, *PERF + LGE+* regions had the lowest MPR values (1.8 ± 0.7). *PERF-LGE-* regions had the highest MPR values (2.5 ± 0.9). *PERF + LGE-* regions had MPR values of 2.1 ± 1. *PERF-LGE+* regions had MPR values of 2.0 ± 0.8. A significant difference was observed between all categories (*p* < 0.0001).

Comparing high-resolution and segmental perfusion quantification results, high-resolution measured significantly lower MPR values in all groups with the exception of PERF-LGE-. Detailed results are also presented in Fig. 3.

### Ischemic burden measurements

**Fig. 4:**
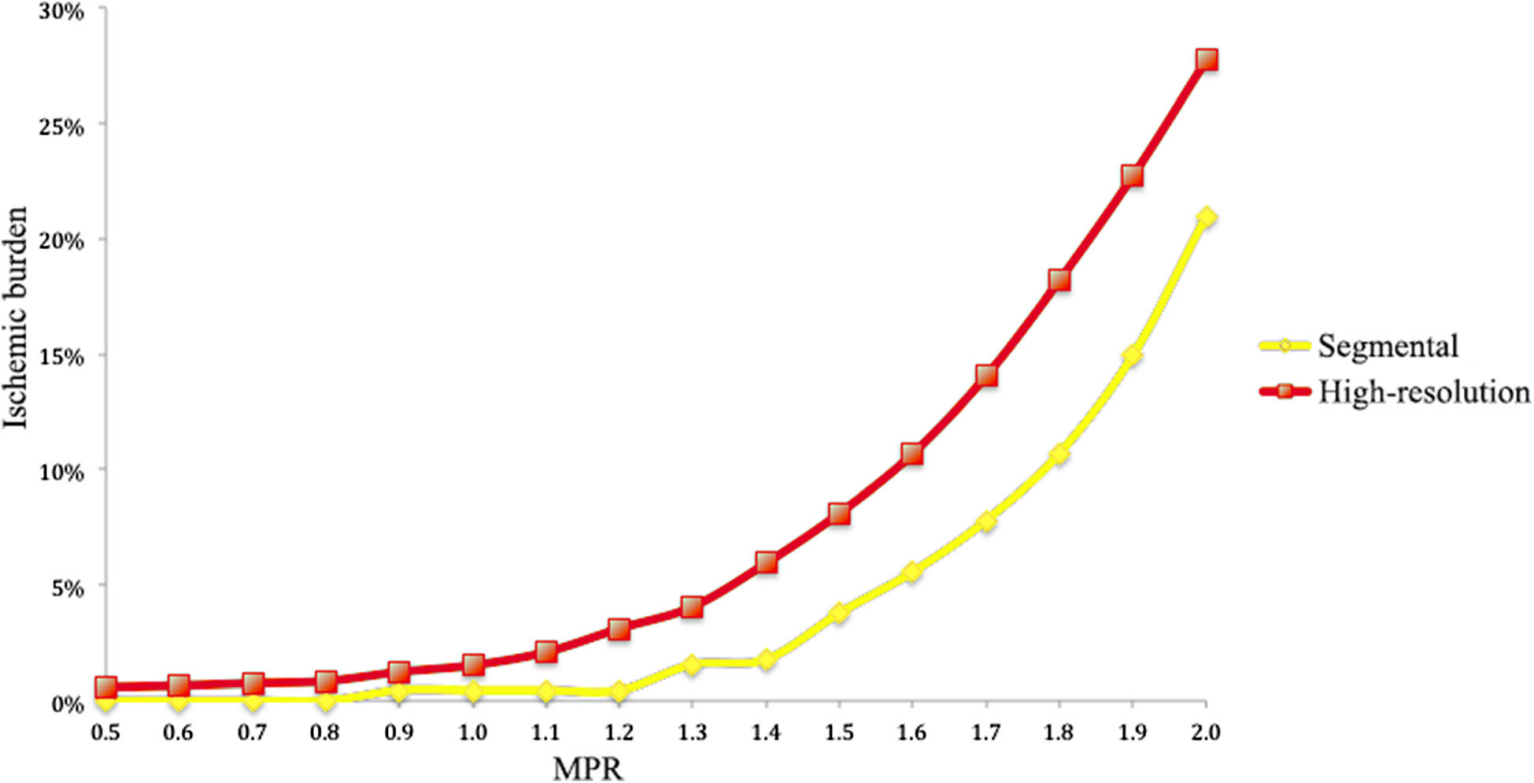
Correlation between the different myocardial perfusion reserve (MPR) thresholds and the percentage of ischemic burden for segmental and high-resolution perfusion quantification

Total LV ischemic burden for segmental and uncorrected high-resolution perfusion quantification are shown in Fig. 4. High-resolution quantification proved more sensitive to the detection of myocardial ischemia in comparison with segmental analysis, with larger ischemic burden detected at all MPR thresholds (*p* < 0.001).

**Fig. 5:**
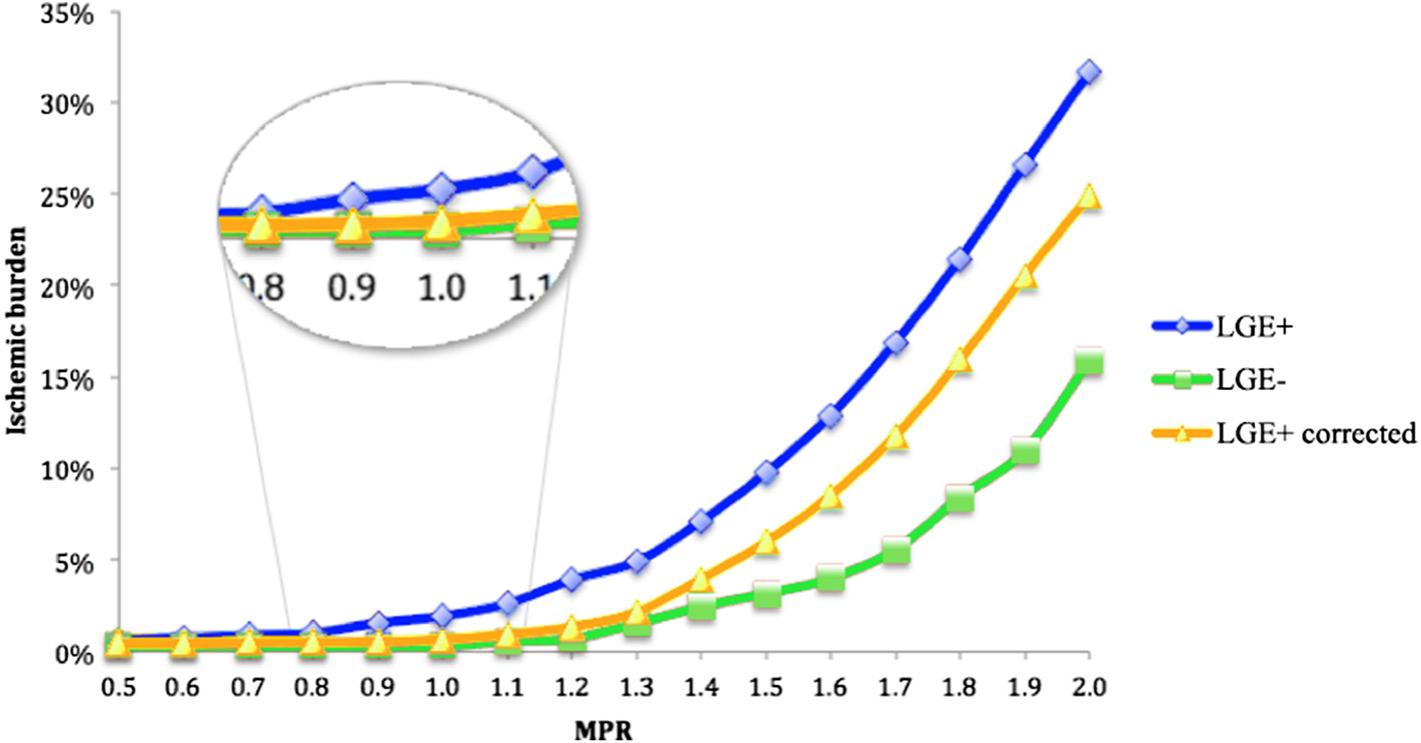
Correlation between the different myocardial perfusion reserve (MPR) thresholds and percentage of ischemic burden for high-resolution perfusion quantification of patients with a visual perfusion abnormality with and without including areas with overt late gadolinium enhancement (LGE), and in patients without LGE

**Fig. 6:**
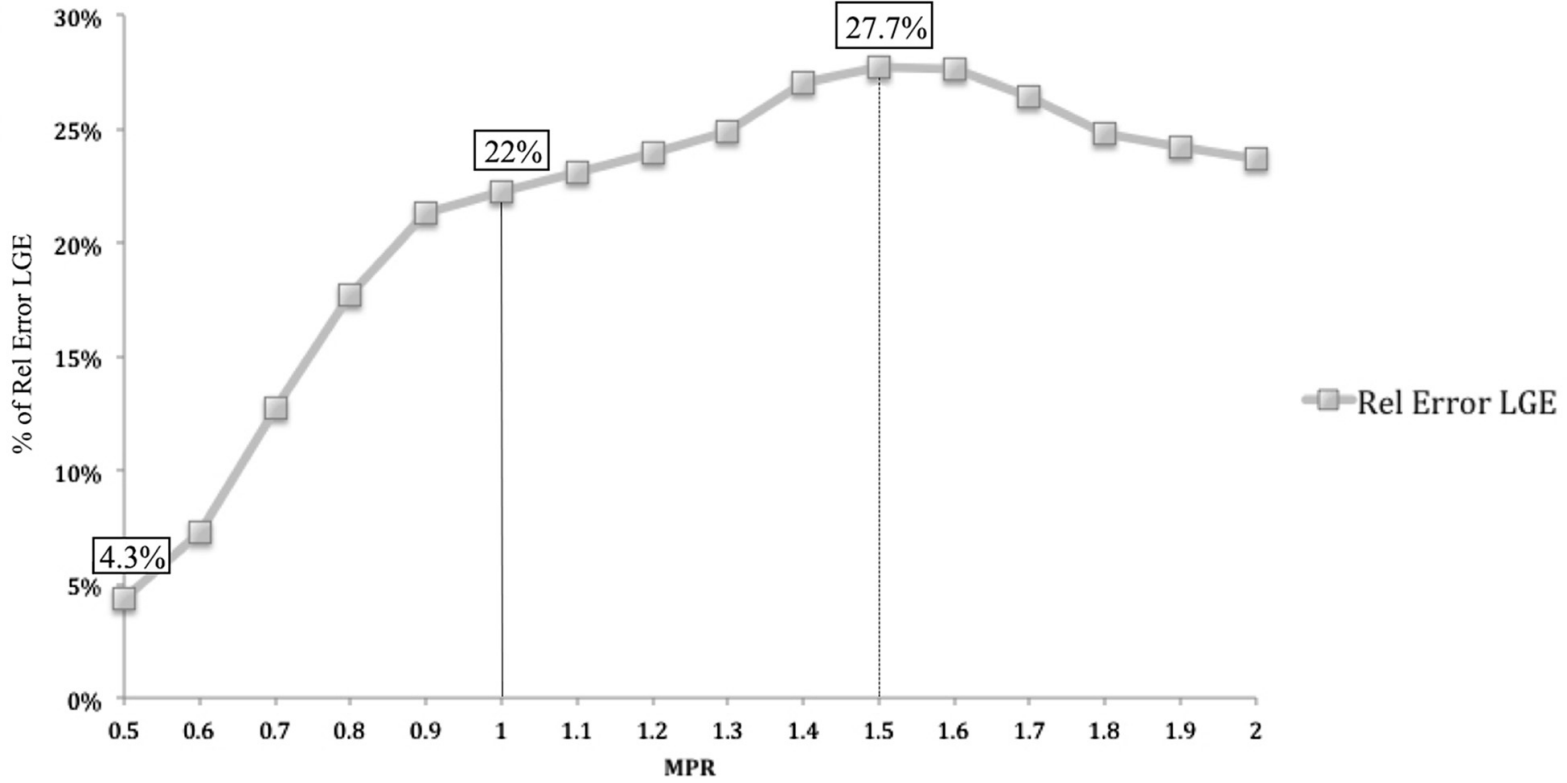
Correlation between the different myocardial perfusion reserve (MPR) thresholds and relative error due to the inclusion of overt scar in the high-resolution perfusion analysis LGE: late gadolinium enhancement

**Fig. 7:**
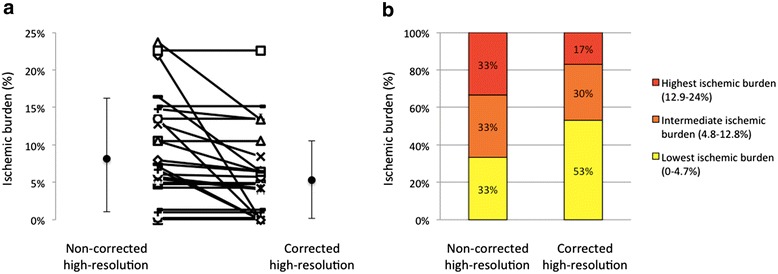
**a** Non-corrected and corrected ischemic burden. Individual cases and average and standard deviation are shown. **b** Tertiles of non-corrected high-resolution ischemic burden and recategorization after correction for late gadolinium enhancement (LGE)

The LV ischemic burden on high-resolution quantification in LGE+ and LGE- patients is shown in Fig. 5. For LGE+ patients, both corrected and uncorrected ischemic burden percentages are shown. LGE+ patients had a significantly higher ischemic burden in comparison to LGE- patients at all considered MPR thresholds (*p* = 0.01). Correction of the results by exclusion of regions with overt LGE caused a significant reduction of the measured ischemic burden at all thresholds (*p* = 0.04). Despite this adjustment however, LGE+ patients had a higher ischemic burden in comparison to LGE- patients for all MPR thresholds above 1.3 (*p* = 0.04). In order to examine the effect of overt LGE on measured ischemic burden at different MPR thresholds, the percentage difference (or relative error) of ischemic burden between corrected and non-corrected high-resolution perfusion maps was calculated, using uncorrected results as the normalization factor (Fig. 6). The relative error in ischemic burden measurements increased steeply between an MPR threshold of 0.5 and 1 (relative error 4.3 and 22 %, respectively), increased at a slower rate between an MPR of 1 and 1.5 (relative error 27.7 %) and was stable or slightly decreased for MPR > 1.5. The comparison between uncorrected and corrected ischemic burden per patient is shown in Fig. 7. The patients were grouped by tertiles of ischemic burden before correction by LGE (lowest ischemic burden 0–4.7 %; intermediate ischemic burden 4.8–12.8 %; highest ischemic burden 12.9–24 %). When results were corrected by the presence of LGE, a total of 9/30 patients (30 %) were recategorised to a lower tertile of ischemic burden. Specifically, 4 patients were re-classified from the intermediate ischemic burden group to the lowest ischemic burden group; 2 patients were re-classified from the highest ischemic burden group to the lowest ischemic burden group; 3 patients were reclassified from the highest ischemic burden group to the intermediate ischemic burden group.

**Fig. 8:**
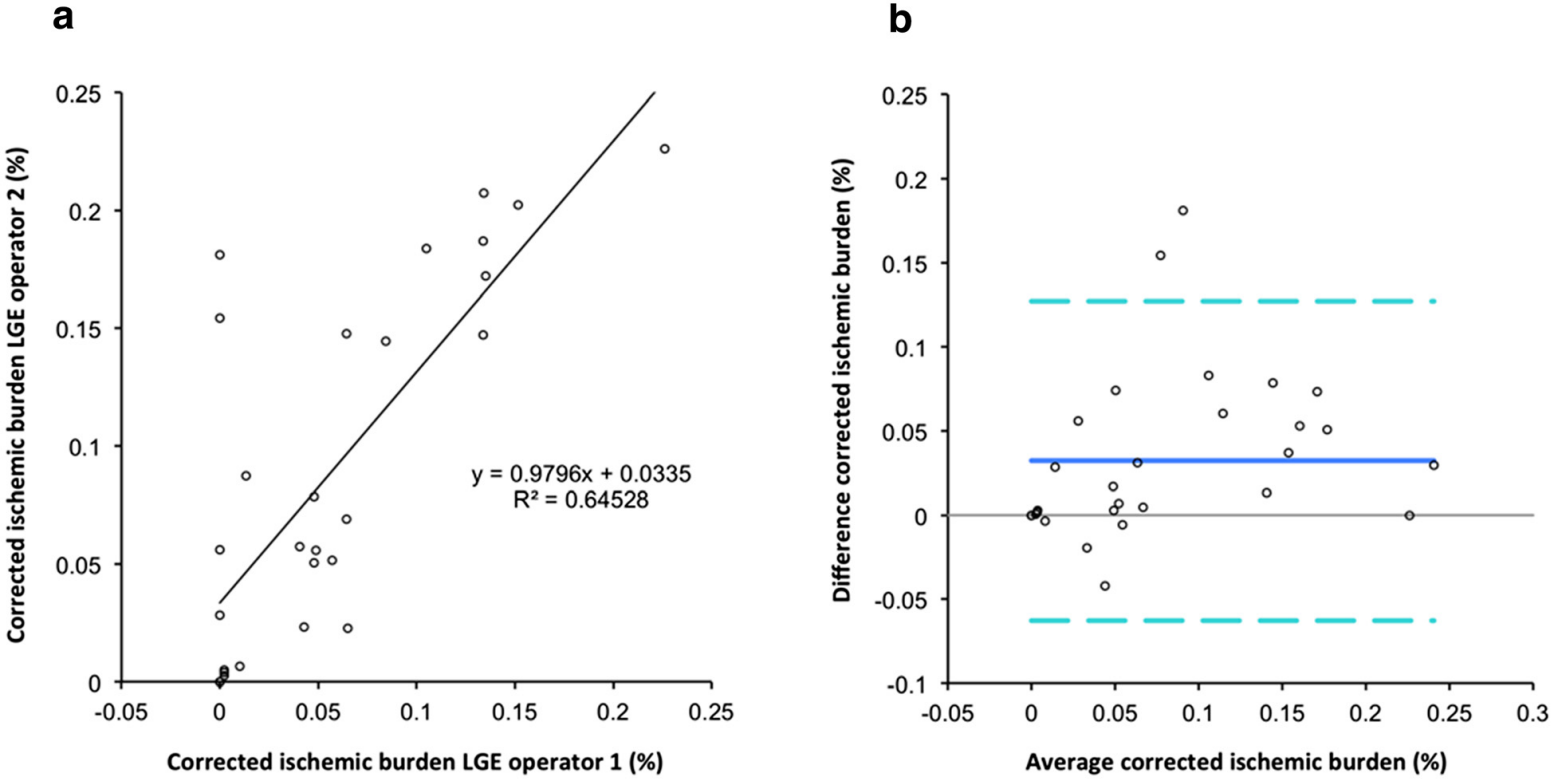
Impact of the inter-observer variability of late gadolinium enhancement (LGE) analysis on corrected ischemic burden measurements. **a** Pearson’s analysis. **b** Bland-Altman graph LGE: late gadolinium enhancement

Corrected ischemic burden proved to be robust to inter-observer variability of LGE measurements. Reproducibility results are shown in Fig. 8 for a MPR of 1.5.

## Discussion

The main findings of this study are: 1) combined CMR high-resolution quantitative assessment of myocardial perfusion and LV fibrosis is feasible in patients with HCM; 2) high-resolution quantitative perfusion assessment preserves the resolution of the original imaging data, is more sensitive than segmental quantitative analysis for ischemia and allows for correction of perfusion data for LGE; 3) LV scar has a significant confounding effect on ischemic burden measurements; 4) 30 % of patients are re-classified to a lower tertile of ischemic burden if high-resolution perfusion maps are corrected by LV scar; 5) patients with LGE have larger ischemic burden in comparison with patients without LGE. This difference is maintained also after correction of high-resolution perfusion maps by LV scar, possibly indicating more severe disease.

Microvascular dysfunction is an important feature of HCM and can be diagnosed non-invasively by stress perfusion CMR [10, 12], where “perfusion abnormalities” have been interpreted as a surrogate marker of microvascular ischemia. This is thought to be an independent predictor for the development of LV dysfunction, LV adverse remodelling and adverse prognosis [8].

High-resolution perfusion CMR quantification has been proposed as an accurate method to assess microvascular ischemia in patients with HCM. Despite the lower signal-to-noise ratio, high-resolution perfusion CMR quantification is capable of very accurate estimates of MPR, as demonstrated previously by our group and by others [20, 21, 35, 36].

Current results confirm the feasibility of this approach and demonstrate the potential to perform a combined assessment of quantitative perfusion and LGE maps and measure the effect of the presence of overt scar on non-invasive measurements of ischemic burden.

It has previously been proposed that high-resolution perfusion quantification could be more sensitive for the diagnosis of microvascular ischemia by avoiding spatial averaging of the data or arbitrary assumptions on the distribution of ischemia [12]. To the best of our knowledge however, our study is the first presenting a comparison between ischemic burden measurements obtained by segmental and high-resolution approaches and demonstrating evidence for this assumption. Previous reports based their results on subsegmental ROIs drawn on perfusion maps and no results were given in terms of ischemic burden or comparison between segmental and high-resolution perfusion analysis [12]. Our results demonstrate that high-resolution quantitative perfusion assessment is more sensitive than segmental quantification both in terms of MPR (Fig. 3) and ischemic burden (Fig. 4), providing differentiation between segments with different combinations of LGE and visual perfusion abnormalities. No significant differences between high-resolution and segmental MPR results were observed for normal segments (PERF-LGE-), where less MPR variability would be expected.

The presence of LGE related perfusion abnormalities at rest in patients with HCM is well described in the literature [16–19]. An effect of scar on perfusion results is also expected from histologic studies that have shown that a reduction of vascularity is associated with scar [37–39]. While an association between microvascular dysfunction and LGE has been shown before [10], specific interaction between overt LV scar and high-resolution perfusion quantification has not previously been investigated. Our results demonstrate for the first time the potential confounding effect of LGE on MPR measurements. This finding is potentially of high relevance, since previous reports suggesting an independent role of “severe microvascular ischemia” in the prediction of events have not taken into account the coexistence of LV fibrosis and its potential interaction as covariate in the risk prediction model. Indeed, “severe microvascular ischemia” was defined in these studies as areas with extremely reduced MPR values ≤ 1.1 [7] or ≤1 [12]. However, adenosine mimics metabolic vasodilatation and a degree of hyperemia is expected also in patients with microvascular dysfunction, even though of reduced magnitude in comparison with normal subjects. Therefore, no areas of MPR < 1, suggestive of an opposite hemodynamic effect, are expected, unless the presence of LGE and a reduction of vascularity are taken into account. This is supported by our results, as areas with MPR < 1 were also seen on our uncorrected high-resolution MPR maps. However, these areas corresponded almost entirely with areas of overt scar. Fig. 6 represents the relative MPR error caused by including areas with severe fibrosis in the ischemic burden and demonstrates that the majority of the error was introduced for MPR values below 1. No further increase in the relative MPR error was observed above MPR values of 1.5, which has been shown before to correlate well in patients with CAD with invasive reference standards for the diagnosis of ischemia, such as FFR [40]. Therefore, the described association between microvascular dysfunction and prognosis could be partially explained by the coexistence of LV fibrosis, which is a known independent predictor of risk [41].

The importance of accounting for LV scar in the assessment of the ischemic burden is further supported by the finding that almost one patient in three was re-assigned to a lower tertile of ischemic burden as result of the correction. (Fig. 7).

Another important finding of our study is that patients with LGE had larger ischemic burden in comparison with patients without LGE and that correction of high-resolution perfusion maps by LV scar did not fully eliminate this difference. This finding could be interpreted in a number of ways. It might indicate more severe disease and microvascular dysfunction in patients with LGE. It might also be explained by technical factors. We decided to correct high-resolution perfusion maps for LV scar defined as regions with signal >6SD above the average of remote normal tissue. Selecting lower thresholds would result in the exclusion of larger areas of fibrotic myocardium and in a smaller difference between LGE- and LGE+ patients. Given the complexity of histological validation on significant number of cases, we feel that this should be evaluated against outcomes in future studies. Corrected ischemic burden results proved in the current study to have a good reproducibility when LGE was quantified by different operators.

### Limitations of the study

This study enrolled a limited number of patients referred on clinical grounds. The prevalence of perfusion abnormalities therefore does not represent the entire population of patient with HCM but rather a group of patients with HCM and suspected microvascular angina. None of the patients included in this study had severe hypertrophy (>30 mm) and therefore we cannot comment on the effect of fibrosis correction in this subgroup.

## Conclusion

This study demonstrates the feasibility of combined high-resolution fibrosis and perfusion quantification and the potential of this method to provide more accurate information on the ischemic burden. Further studies are awaited regarding the impact of this novel approach on risk stratification.

## Abbreviations

HCM: hypertrophic cardiomyopathy
SCD: sudden cardiac death
LV: left ventricle
PET: positron emission tomography
SPECT: single photon emission computed tomography
CMR: cardiac magnetic resonance
LGE: late gadolinium enhancement
SI: signal intensity
SD: standard deviations
ROI: region of interest
MBF: myocardial blood flow
MPR: myocardial perfusion reserve
PERF: perfusion abnormalities
